# What services, interventions and support are available for People with HIV in England to manage their overall health and wellbeing? A scoping review

**DOI:** 10.1111/hiv.70041

**Published:** 2025-05-07

**Authors:** Howell T. Jones, Lucy Lynch, Tristan J. Barber, Meriel Rattue, Laura J. Waters, David Asboe, Angeline Walker, Ian Williams

**Affiliations:** ^1^ Ian Charleson Day Centre Royal Free London NHS Foundation Trust London UK; ^2^ Institute for Global Health, UCL London UK; ^3^ UK Health Security Agency London UK; ^4^ School of Primary care, Population sciences, and Medical education (PPM), Faculty of Medicine University of Southampton Southampton UK; ^5^ Terrence Higgins Trust London UK; ^6^ Bloomsbury Clinic, Mortimer Market Centre Central and North West London NHS Foundation Trust London UK; ^7^ Kobler Clinic Chelsea and Westminster NHS Foundation Trust London UK; ^8^ NHS England London UK

**Keywords:** ageing, AIDS, England, frailty, HIV, long‐term condition, multimorbidity, services

## Abstract

**Objectives:**

The average age of a person living with HIV in high‐income countries is increasing, as are rates of multimorbidity and frailty. To meet these needs, existing services must adapt. This review aimed to identify the existing literature on what services are available to undertake long‐term condition management (LTCM) for People with HIV in England.

**Methods:**

A scoping review employing the Arksey & O'Malley's methodological framework was performed. Seven databases were searched most recently in October 2024 for studies describing services, interventions, or support for People with HIV in England to manage their overall health and wellbeing. Study inclusion was not limited by year of publication. Narrative reviews were excluded. Two reviewers independently performed data extraction using predetermined criteria, followed by a descriptive analysis.

**Results:**

Forty publications were identified with six key areas where LTCM was addressed: HIV services, secondary care services, primary care, palliative care, peer support, self‐management, and specialist services, suggesting that currently no service can undertake LTCM alone.

**Conclusions:**

If LTCM for People with HIV is to expand outside of HIV services, then additional HIV training is required for healthcare professionals with a focus on reducing stigma. Peer support should be at the forefront, and People with HIV should be involved in the assessment of need, design, and evaluation of services. There is a scarcity of high‐level evidence, which justifies the need for further research and ongoing service evaluation to identify the optimal model(s) to ensure effective, equitable, and cost‐effective care.

## INTRODUCTION

Long‐term conditions are physical or psychological problems that require ongoing management over a period of years [1]. They are incurable but can be managed with medication or other therapies [1]. A person‐centred approach is central to long‐term condition management (LTCM) [2]. Effective antiretroviral therapy (ART) means HIV is a manageable long‐term condition with a life expectancy comparable with people without HIV [2, 3].

**FIGURE 1:**
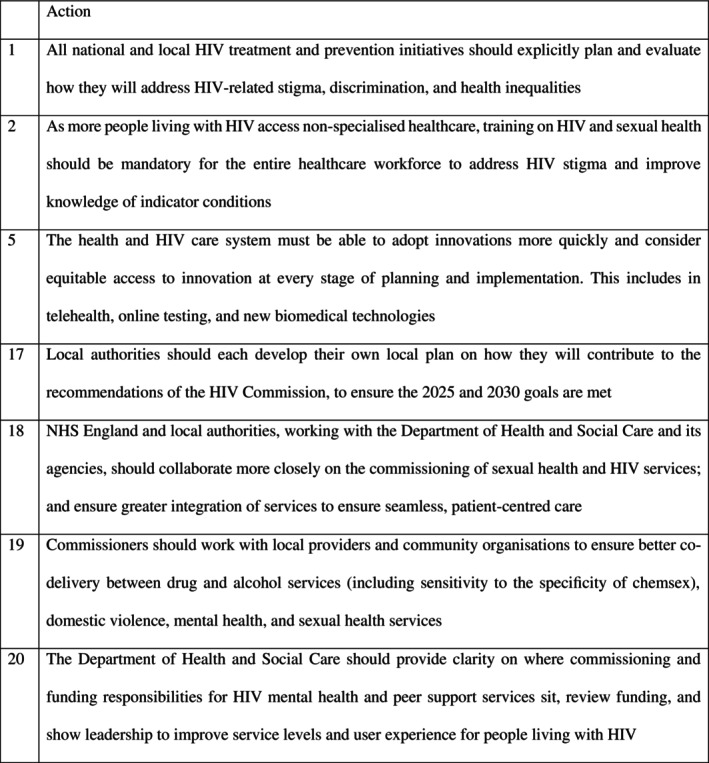
Actions from the HIV commission relevant to this review [1].

People with HIV may experience stigma, discrimination, and inequality, which can negatively impact their physical or mental health [4, 5]. The HIV Commission highlights the importance of working in partnership to manage HIV over a person's life course by pooling resources to share skills, experience, and best practice (Figure 1) [2]. Insufficient collaboration leads to competing priorities, duplication, and service gaps [2]. Local leadership is required to combine resources to maximize the provision of effective joined‐up services [4, 5].

Older people account for an increasing number of HIV service users, with approximately half of all people who accessed HIV services in England in 2021 being over 50 years old and an expectation that this will be as high as 70% by 2030 [6, 7] These changes will have implications for the delivery of healthcare services, which are currently fragmented across primary, secondary, and tertiary care [7, 8].

HIV services will have to adapt to manage new conditions [2]. Firstly, multimorbidity, which is the coexistence of two or more long‐term health conditions including physical or mental health conditions, learning disabilities, alcohol or substance use, sensory impairment, or symptom complexes such as frailty [9]. Frailty is the age‐related loss of in‐built reserve, leaving a person vulnerable to dramatic, sudden changes in health triggered by seemingly small events such as a minor infection or a change in medication [10, 11]. Both these phenomena may result in polypharmacy, defined as taking five or more medicines, which in turn can have a negative impact on a person's health [12].

There has been limited commissioning support for LTCM associated with HIV in England. Guidance is required to enable commissioners to identify strategies to best support People with HIV across their lifetime, necessitating the need to establish the current landscape on the topic.

This review aims to consolidate the literature on what services, interventions, and support are available for People with HIV in England to manage their overall health and wellbeing.

## METHODS

The LTCM sub‐group of the national HIV Clinical Reference Group acting as a specialty advisory committee for NHS England was asked to conduct a scoping review to understand the current landscape in England. The methodological framework developed by Arksey and O'Malley was used where first a research question is identified, followed by identifying relevant studies, study selection, data charting, and summarizing results respectively [13]. Reporting followed the Preferred Reporting Items for Systematic Reviews and Meta‐Analyses (PRISMA) Extension for Scoping Review checklist [14].

A comprehensive search of published research was conducted in April 2021 with seven computerized databases (AMED, CINAHL, EMBASE, EMCARE, HMIC, Medline, and PsychINFO) accessed using synonyms of the keywords ‘HIV’, ‘service’, ‘England’, and ‘chronic illness’ or ‘long‐term condition’. The grey literature was also searched (‘Proquest Dissertations & Theses’ and ‘OpenGrey’). Reference lists of identified papers were hand‐searched for additional results. The search was updated regularly, most recently in October 2024. The full search strategy is outlined in Appendix 1.

The research question was ‘What services, interventions, and support (from the public, private or third sector) in England are available for adults living with HIV to manage their overall health and wellbeing over the long term?’

Following removal of duplicates, two reviewers (HTJ and TJB) independently assessed the titles and abstracts for eligibility. Potentially eligible full texts were then reassessed against the eligibility criteria by both reviewers. A third reviewer was available should differences in opinion arise.

### Eligibility criteria

#### Population

Any studies exploring strategies for LTCM in adults living with HIV were incorporated, including descriptive studies without control populations, or articles describing healthcare models. Studies specific to the paediatric population were excluded unless they concerned adolescents transitioning to adult services. No other limitations based on population characteristics were applied.

#### Concept

Any publications reporting strategies for LTCM in People with HIV were included regardless of their primary aims.

#### Context

Only studies relating to services in England were eligible for inclusion.

#### Study type

This scoping review was not limited to peer‐reviewed primary research articles or systematic reviews and also includes conference abstracts and grey literature. Narrative reviews were excluded, but their references were examined for additional eligible studies. There was no limitation on the year, but only publications in English were included.

The reviewers determined what data was to be extracted prior to data charting to maintain consistency. Extracted data included: title, authors, publication year, publication type, location in England, study aims, sample size, and demographics, study design, services identified, and key findings. Extracted data was examined by both reviewers for clarity and reliability. Extracted information was tabulated according to the categories outlined above, with a descriptive analysis of extracted information performed and presented narratively.

## RESULTS

**FIGURE 2:**
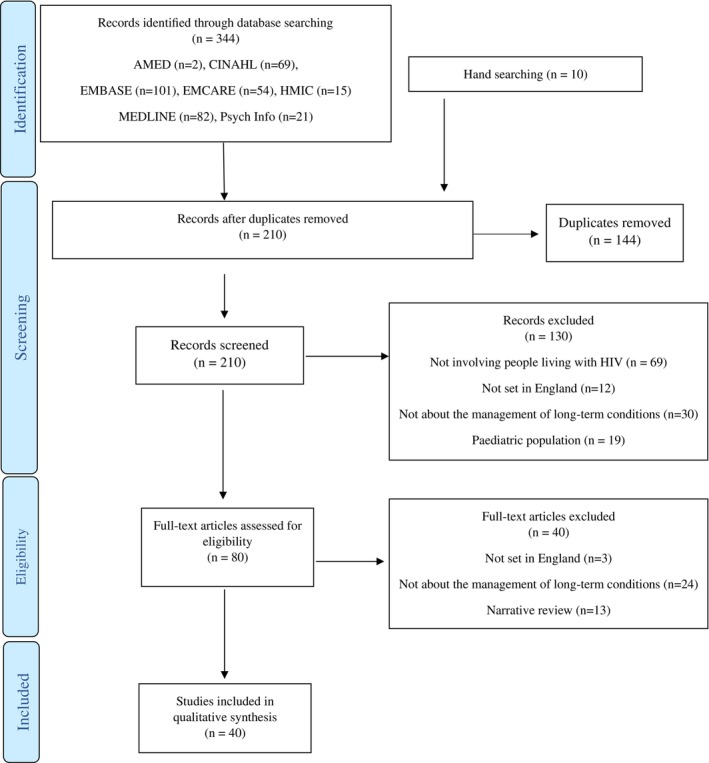
Preferred Reporting Items for Systematic Reviews and Meta‐Analyses (PRISMA) diagram [13].

The final database search yielded 344 results, which were reduced to 200 after removal of duplicates, with an additional 10 articles identified through hand‐searching. Screening records by title and abstract resulted in 80 full‐text articles or conference abstracts being reviewed, with 40 meeting the inclusion criteria. No further studies were identified from the grey literature. The full PRISMA flow diagram is displayed in Figure 2 [14].

The 40 identified studies were published between 1993 and 2024, with 8 (20%) being published within the last 5 years and 22 (55%) within the last 10 years (Appendix 2) [15, 16, 17, 18, 19, 20, 21, 22, 23, 24, 25, 26, 27, 28, 29, 30, 31, 32, 33, 34, 35, 36, 37, 38, 39, 40, 41, 42, 43, 44, 45, 46, 47, 48, 49, 50, 51, 52, 53, 54]. Twenty‐seven publications (68%) were primary research articles, 12 (30%) were conference abstracts, and 1 (3%) was a report [15, 16, 17, 18, 19, 20, 21, 22, 23, 24, 25, 26, 27, 28, 29, 30, 31, 32, 33, 34, 35, 36, 37, 38, 39, 40, 41, 42, 43, 44, 45, 46, 47, 48, 49, 50, 51, 52, 53, 54]. Cross‐sectional and qualitative methodology were each used in 17 (42%) of the studies, 3 (8%) were cohort studies, 2 (5%) were mixed‐methods studies, and 1 (3%) was a systematic review [15, 16, 17, 18, 19, 20, 21, 22, 23, 24, 25, 26, 27, 28, 29, 30, 31, 32, 33, 34, 35, 36, 37, 38, 39, 40, 41, 42, 43, 44, 45, 46, 47, 48, 49, 50, 51, 52, 53, 54]. The total number of People with HIV included (where reported) was 17 703 [15, 16, 17, 18, 19, 20, 21, 22, 23, 24, 25, 26, 27, 28, 29, 30, 31, 32, 33, 34, 35, 36, 37, 38, 39, 40, 41, 42, 43, 44, 45, 46, 47, 48, 49, 50, 51, 52, 53, 54].

### Seven key areas of care were identified from the literature (Figure 3)

**FIGURE 3:**
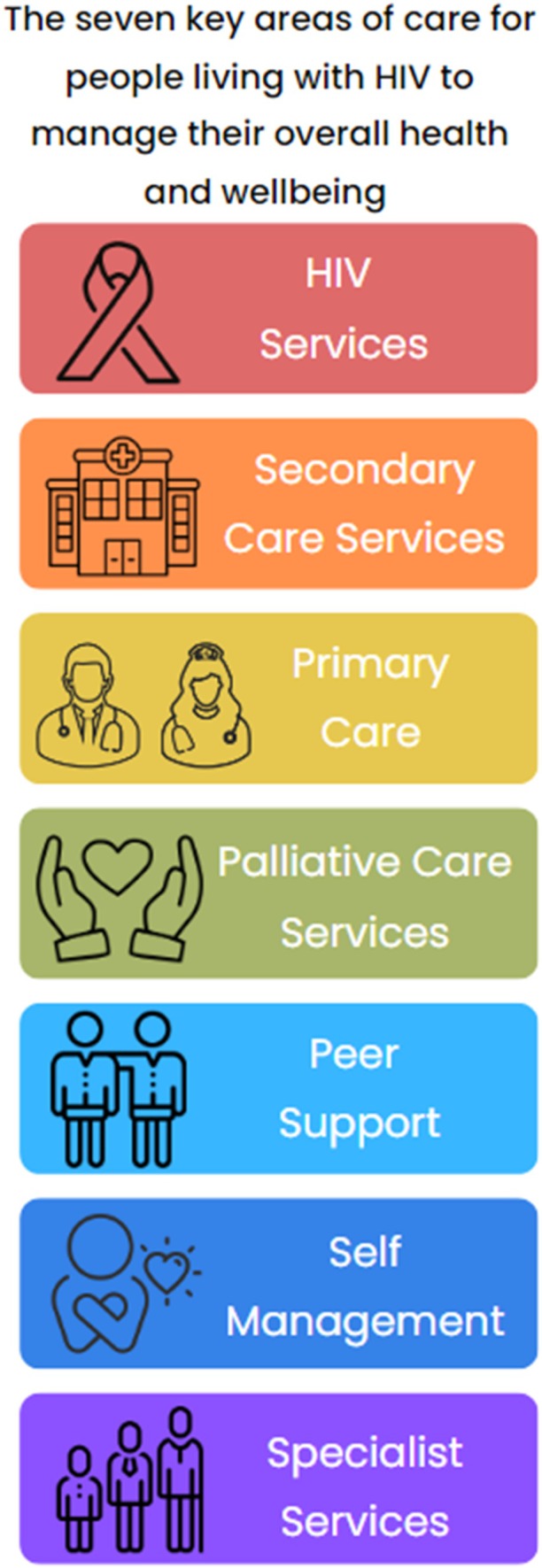
Key areas of care to help people living with HIV manage their overall health and wellbeing.

#### HIV services

The role HIV services play in LTCM was explored in 14 studies [15, 17, 18, 19, 27, 36, 37, 39, 40, 44, 45, 48, 50, 51]. People with HIV prefer receiving LTCM within their HIV service, especially if they experience uncertainty about their future health [36, 39, 44, 45]. In England, some HIV services are already addressing LTCM, focusing primarily on cardiovascular and bone health, with high user satisfaction rates [15, 37, 48]. In a 2016 cohort study in London, 26% (*n* = 99) of hospital admissions of People with HIV were related to LTCM [50]. A 2018 cross‐sectional study (*n* = 4415) reported that 42% of respondents had unmet LTCM needs, and 62% had unmet social care needs [27]. These themes were echoed in a 2019 qualitative study (*n* = 28) where 95% of participants felt involved in decisions about their HIV care, while only half had their LTCM needs met [48].

How to identify unmet needs remains unclear. A ‘wellness thermometer’ has been trialled at HIV services in Manchester, East Sussex, and Milton Keynes leading to improved service user‐service provider relationships and increased service user satisfaction [19]. A 2017 national survey reports that HIV clinicians feel well equipped to support People with HIV with LTCM but in cases outside their scope of practice they refer on to primary or secondary care services [18].

Two studies explored how HIV services should be delivered in the future; people with minimal illness/symptom experience may prefer virtual models of care (such as telephone or e‐mail clinics) while those with higher illness/symptom experience are more likely to rely on face‐to‐face interactions [39, 40]. Community‐based satellite HIV clinics within General Practice have reported increased user satisfaction due to improving access through a reduction in travel and tackling stigma [40].

With regards to frailty screening and management, a qualitative study in Brighton identified that People with HIV prefer this to be done within their HIV service with onward referrals to other services only when necessary [51]. This contrasted with the views of HIV healthcare professionals who felt that frailty screening was time‐intensive and they were concerned about labelling younger people as frail due to a lack of services available to support them [51].

While paediatric HIV services were excluded from this review, studies involving the transition of adolescents to adult services were included, highlighting the unique needs of this group, meaning HIV services should have strategies in place to support young people with LTCM during their transition [17].

#### Secondarycare services

The role of existing secondary care services was discussed in 2 studies [32, 48]. Positive Voices, a cross‐sectional survey of People with HIV (*n* = 4400), undertaken in 2017 reported that 77% of respondents had faced difficulties in accessing secondary care services and navigating the National Health Service [48]. Reported barriers to accessing secondary care services for LTCM included clinicians’ perceived lack of knowledge or confidence in treating or prescribing for conditions where HIV is also present [48]. Respondents also felt they would have to educate healthcare professionals about HIV or drug–drug interactions [48]. Stigma, both anticipated or experienced, was also highlighted as a barrier, with 35% of participants fearing being treated differently, 14% having experienced discrimination in healthcare, and 11% having been denied or refused a treatment or a procedure due to their HIV status [48]. Similar findings were described in a qualitative study of people of Black African or Black Caribbean ethnicity in London, where experiences of HIV stigma as well as culturally‐or racially‐based discrimination were identified as barriers to engaging with secondary care services [32].

#### Primary care

Primary care was identified as being well placed to address LTCM for People with HIV and therefore explored in 19 studies [15, 16, 18, 20, 21, 22, 23, 25, 28, 30, 31, 32, 33, 35, 36, 38, 44, 45, 49]. Some people may choose to not share their HIV status with their General Practitioner (GP) due to anticipated stigma or discrimination [4, 5]. Nevertheless, between 81% and 89% of People with HIV in England are registered with a GP and 78% have shared their status [25, 44, 45, 52]. The BHIVA primary care project ‘Shared care: how can we do it?’ reported that the average number of People with HIV on a GP practice list was 35 (range 0–150) which would give an average prevalence of 3.5/1000 [36]. Positive Voices found a mean GP satisfaction rate of 6.9/10 (HIV services 9.3/10) [15]. Following an increased access to ART, GP consultation rates by People with HIV have declined to those of the general population with a rate of 4.2 consultations per year for men and 5.2 consultations per year for women reported in 2005 [21]. A study in primary care reported that 29% (*n* = 873) of respondents had at least one additional long‐term health problem, most commonly hepatitis, mental health conditions, and cardiovascular disease reinforcing the prevalence of multimorbidity in this population [18, 35, 36].

A survey of 124 GPs reported that 93% felt comfortable to deliver LTCM in People with HIV with 60% believing GPs should manage their hypertension, diabetes mellitus and hyperlipidaemia [20]. GPs believe they provide personalized care, local knowledge, 24‐h availability, and a more ‘normal’ environment than a hospital [25, 31, 36]. However, a lack of knowledge around HIV was identified as a barrier to delivering LTCM in primary care [36, 55]. GP engagement may be dependent on their experience local prevalence of HIV and level of morbidity [25, 31]. The impact of the primary care electronic summary care record (SCR) was discussed in two studies with some People with HIV feeling it improves communication and consolidates care while others felt it posed a risk to their confidentiality and lessened their control of who had access to their HIV status [23, 45]. Conversely some people welcomed their status being visible in their record so that their GP could be aware without them having to inform them, which in turn, could reduce stigma [23].

People with HIV feel that GPs do have a role in their care due to their experience in LTCM, but they perceive GPs to have low levels of knowledge about HIV, leading to a lack of confidence in assisting them with LTCM [16, 25, 30, 32, 36, 44, 45, 55]. The most frequently cited strength of GP care was locality [36]. They also feel that community nurses lack the knowledge to support them, while community pharmacists were considered the least helpful health professionals in terms of medication support [16, 28]. People with HIV reported that community‐based mental health services were less likely to understand the impact of HIV on psychological morbidity and were likely to be less empathetic than psychological services within an HIV service, which contrasts with the views of GPs who believe they are well placed to provide support for mental health problems associated with HIV [16, 44, 55].

The BHIVA primary care project identified that the preferred model of care of GPs (*n* = 113) is one of GP‐led care with educational updates by HIV specialists followed by ‘shared care’ [36]. A move to shared care has been considered in several studies with one reporting that 67% (*n* = 124) of GPs were in favour though a proposed barriers was the perception that People with HIV want all of their care including LTCM to be undertaken by HIV specialists [15, 20, 25]. Shared care was not popular with People with HIV due to concerns it could lead to lack of continuity with people reporting that they often see different GPs, whereas they tended to see the same HIV clinician [30, 36, 44]. They also felt that moving care from HIV services to primary care was confusing and associated with poor communication leaving them unclear as to who had responsibility for aspects of their care such as referrals, prescribing, and care coordination [30, 44, 45]. This is particularly true for those with multimorbidity or who have been living with HIV for a long time who reported a preference to maintain their care at a specialist level [45]. Overall, for People with HIV their optimal model of primary care would be to have a GP within their HIV service [36].

Finally, People with HIV have a higher burden of unmet dental needs when compared with the general population (54.9% vs. 32.1%) [22, 33, 38]. A London‐based study (*n* = 51) reported they have also had more difficulty registering with a community dentist (58.8% vs. 18.2%) [22, 33, 38]. Studies found that 68%–74% of people changed their dentist following a diagnosis of HIV and 55%–64% of people had shared their status with their current dentist [22, 33, 38]. The main reasons for not seeing a dentist were concerns around confidentiality or refusal of treatment [38]. This is not unfounded as refusal of dental care based on HIV status was reported as high as 25% (*n* = 69) in a 1998 national study, though this has fallen to 4% in a 2005 Manchester‐based study (*n* = 81), and to 6.2% (*n* = 51) in a 2009 London‐based study [22, 33, 38]. Due to these experiences, People with HIV often reported a preference for hospital‐based dental clinics to manage their long‐term oral health [22, 38].

#### Palliative care services

The role of palliative care services in supporting People with HIV with LTCM was explored in 2 studies [31, 47]. A survey of primary care staff reported that end‐of‐life care was not considered important in the care of People with HIV by any of the doctors sampled and by only 3% of nurses [31]. However, palliative care services can support delivering LTCM and with sensitive advance care planning [47].

#### Peer support

Peer support is central to HIV care though it was only described in 5 studies as the literature tended to focus more on the role of healthcare professionals [27, 32, 43, 47, 53]. Community organization support has been shown to improve people living with HIV's knowledge of LTCM, teach them strategies to improve their health, and support them to access healthcare services [27, 32]. Peer support is also important in preventing loneliness and social isolation [43, 47, 53]. Unfortunately, despite this, in a cross‐sectional study (*n* = 4415) 32% of people reported they did not have access to the level of peer support they desired [27].

#### Self‐management

Self‐management is important for LTCM, with 8 studies exploring its role in the context of HIV [29, 32, 41, 42, 46, 47, 48, 54]. Goal setting, promoting independence, enhancing recovery, recognizing progress, and valuing reassurance from others are key aspects of self‐management [42]. People with HIV may face self‐management problems similar to those of other long‐term health conditions, but they can be supported to manage their own health, leading to a reduction in healthcare usage; one study reported that 95% of participants felt equipped by their HIV service to self‐manage [47, 48].

A study exploring the role of physiotherapists and occupational therapists in assisting People with HIV with self‐management concluded that improved access to rehabilitation services is required [41, 42]. The same study identified that the goals, values, preferences, and expectations may differ depending on a person's age and their length of time living with HIV, which in turn impacts what methods of self‐management are most likely to be effective [42]. Group workshops can support self‐management techniques while also providing opportunities to create relationships between the user, other attendees, and the host organization [29].

Many People with HIV want to be able to access interactive and comprehensive self‐management information online to assist them with LTCM [46]. The Patient Activation Measure (PAM) tool, which assesses people's knowledge, skill and confidence in managing their own health to identify those most likely to benefit from self‐management techniques has been shown to be effective when used within HIV services [54]. It is important that resources consider people's cultural traditions and financial constraints, as these have been identified as barriers to self‐management, especially in the context of interventions such as diet and exercise [32].

#### Specialist services for people ageing with HIV

The last strategy for supporting People with HIV with LTCM are specific services created with that aim in mind, hereby referred to as ‘specialist services’. These were discussed in 5 studies with 3 distinct models described [18, 24, 26, 34, 52]. A 2017 survey of English HIV services identified that at that time only two specialist services existed, and acknowledged that instead the majority of HIV services conducted multidisciplinary team meetings where LTCM could be discussed, be it without input from allied health professionals or other medical specialities [18]. The majority (64%) of respondents did not support the need for specialist services with 13% uncertain and only 23% in support [18]. Those who did not support the need did so as they felt with the majority of their service users being over 50 years old addressing LTCM was the standard of care [18].

The first model described was ‘virtual clinics’ such as the ‘HIV in Later Life Virtual Clinic’ at Homerton hospital in London [24]. Launched in 2017, virtual reviews of health records of people aged over 50 years with regards to cardiovascular, renal and bone health with 334 people assessed over the initial 8 months, with improvements to cardiovascular disease risk assessment (93.2% vs. 49.2%) and increased identification of those who required modification of ART or a DEXA scan [24].

The second model was ‘in‐person reviews by HIV clinicians’ such as the ‘Over 50's' clinic at Chelsea and Westminster hospital in London [52]. This service has led to improvements in the overall health of the attendees, particularly in the context of cardiovascular and bone health, with high levels of attendee satisfaction [52].

The final model was one offering a ‘multidisciplinary approach’ such as the ‘Silver Clinic’ in Brighton and the ‘Sage Clinic’ in London with both offering assessments by several healthcare professionals including HIV clinicians, geriatricians, nurses, and specialist pharmacists with the Sage clinic having the addition of physiotherapists and occupational therapists [26, 34]. The Sage clinic identified a high prevalence of frailty in attendees (83%) alongside 18 discrete issues associated with ageing, with the most common being affective symptoms (51%), memory loss (37%), and falls (29%) [26]. The Silver clinic also identified high levels of frailty and low mood amongst its cohort and was well received by the attendees [34]. None of these services have currently reported long‐term outcomes or data regarding cost‐effectiveness [18, 24, 26, 34, 52].

#### Recommendations

Through these seven areas, five key recommendations have been identified:

1. No service can undertake LTCM for People with HIV alone, so a ‘collaborative care’ model is recommended.
2. All healthcare services should place importance on tackling HIV stigma prior to the redirection of care for people living with HIV.
3. Peer support is paramount and should be central to all services.
4. People with HIV should be involved at all stages of service design and evaluation.
5. More focus should be placed on service evaluation to grow the literature base.

## DISCUSSION

People with HIV have varied experiences and heterogenous views about how to meet their diverse needs. The responsibility for LTCM in People with HIV cannot be left to HIV services alone, but currently both primary and secondary care services lack experience in the management of this group [15, 16, 18, 20, 21, 22, 23, 25, 28, 30, 31, 32, 33, 35, 36, 38, 44, 45, 48, 49]. It is also important to understand the legacy of the history and context of HIV care provision [20, 25, 30, 31, 36, 44, 45, 56]. The BHIVA primary care project highlighted that there were mixed views on the concept of ‘shared care’ by both GPs and People with HIV [36]. It may be preferable, instead, to adopt a ‘collaborative care’ approach to ensure that the specific needs of this group are recognized while drawing on the expertise of other specialists such as geriatricians, and considers the ease of access to care in the local community through primary care [57, 58]. This would allow flexibility to determine decisions at both a local and individual level rather than a strict division of care to formulate an individualized care plan [36]. This is particularly important for people living in rural areas who may have to travel long distances to access HIV care, those who are with multimorbidity, and those who have been living with HIV for many years [36, 45, 59]. Nevertheless, it remains likely that this population will either need to transition to or start accessing existing secondary care services despite it being unclear if these services are currently able to manage their overall health [32, 56].

With rising rates of frailty and multimorbidity, the European AIDS Clinical Society recommends frailty screening for older People with HIV, while the British HIV Association supports incorporating geriatricians into their care where needed [11, 56, 60, 61, 62]. However, any changes to existing care models must be undertaken methodically, as rapid changes to existing care models can lead to fragmentation, disengagement, and worse health outcomes [63, 64].

It is also likely that some routine HIV care will be moved to primary care, and while most GPs agree with this, many lack the knowledge, experience, or confidence around supporting People with HIV [36, 44, 55]. While palliative care services may not be relevant to everyone, their expertise in holistic care may be useful in certain scenarios such as chronic pain management [7, 65, 66]. The views of People with HIV should be sought before reallocating responsibility to primary care [25, 30, 36, 44, 45]. The evidence shows that moving care for other long‐term conditions, such as type two diabetes mellitus, from secondary to primary care may reduce healthcare costs at the individual level, but that savings may be offset by increases in overall service volume and loss of economies of scale [67, 68]. Meanwhile, the transfer to primary care does not guarantee improvements in cost‐effectiveness, or in acceptability, accountability, equity, or responsiveness [69].

However, before any changes to LTCM approaches are implemented, HIV stigma and discrimination, both real and perceived, must be addressed, making this a priority for all existing healthcare services accessed by People with HIV [70, 71]. This appears to be especially true for dental services [22, 33, 38]. Stigma leads to worse health outcomes due to its impact on adherence with ART, engagement with care, sharing of status, incidence of depression, and life expectancy [4, 72, 73, 74]. Education about HIV is essential for all healthcare professionals to begin to reduce stigma and improve attitudes towards People with HIV, with interventions targeted at the individual, environmental, and policy levels [5, 56].

Though underrepresented in the literature, peer support remains paramount in managing the overall health and wellbeing of People with HIV and should be central to any service [27, 32, 43, 47, 53]. Peer support has been shown to be effective in improving health outcomes in a variety of settings and for both physical and mental health conditions [75, 76, 77]. In the context of HIV, peer support has been shown to improve concordance with ART, retention in care, sexual health, mental health, and quality of life, while reducing stigma [78, 79, 80]. Therefore, as the needs of the HIV population in England change, the role of peer support must adapt, with one area of unmet need being LTCM [27, 32, 43, 47, 53].

Involving People with HIV in needs assessments, research, service design, and evaluation is critical to improving outcomes [2, 81]. Involving service users in healthcare design is associated with increased satisfaction and service usability along with improvements in overall health and wellbeing [82, 83, 84]. Therefore, People with HIV should be involved at the conception, design, and evaluation of all existing and new services developed to support them with LTCM. The optimal model for this remains unclear though there has been increased focus on digital platforms as a result of the Covid‐19 pandemic [11, 58, 85]. This work reports the positive impact virtual clinics can have for LTCM as they may help address the inequality people living in rural areas face for both HIV care and LTCM [24, 59, 86]. However, the experiences of digital platforms during the Covid‐19 pandemic by both HIV service users and providers were mixed [11, 58, 85]. This suggests again that there is not a ‘one size fits all’ solution with evidence suggesting it may impact the ‘service user‐service provider’ relationship while not routinely reducing the cost of care delivery despite a reduction in carbon footprint [87, 88, 89]. It is also important to consider that People with HIV may experience widening health inequalities as an unintended consequence of using technological solutions if they are digitally excluded [90].

Finally, further research into the best model for LTCM for this population is required. Establishing this would most robustly be done via randomized clinical trials or observational studies comparing models such as virtual versus in‐person care [24, 26]. Studies should also consider the location, i.e., primary versus secondary care, with concepts such as HIV specialists working in community clinics or GPs supporting care from within an HIV service considered [36, 40]. These, however, would be costly, so in the interim it is important to ensure there is a sustained focus on service evaluation [91, 92]. The literature shows that specialist services have been well received by users and successful in improving their overall health [26, 34, 52]. However, while positive results are presented in the short term, limited data exist about long‐term effectiveness with regards to cost savings or health outcomes [26, 34, 52]. Ongoing service evaluation is necessary to ensure interventions lead to improved outcomes in both the short and long term, with a specific focus on cost‐effectiveness and user satisfaction, with current operating models evaluated prior to replication elsewhere [92]. Without this, changes to existing strategies could be expensive yet ineffective, as demonstrated in the recent evaluation of the Acute Frailty Network, which also aimed to address LTCM in the wider older population [11, 58, 60, 91, 92, 93].

Due to the proposed question, this review is narrative in nature and therefore does not allow the formation of a meta‐analysis [94]. As this is a scoping review, its purpose was limited to gathering evidence without critically appraising the quality of the studies included [70]. It is strengthened by the inclusion of conference abstracts and grey literature by consolidating all of the current information and reducing the impact of publication bias [95, 96]. Only manuscripts published in English were included; though as the focus was on care in England, the impact of this is likely negligible [97]. This review was commissioned to focus on care in England, but as patterns of health in People with HIV are similar across high‐income countries, there will be relevance to those working internationally due to the paucity of synthesized data on the topic [60, 98, 99]. However, the impact of differing healthcare systems cannot be underestimated, which may limit generalizability and supports the need for similar reviews to be conducted internationally [60, 98, 99]. The focus on adults means this review is not applicable to children, with further research required for this population [100]. Finally, the studies included in this review often considered the wider HIV population, with groups such as women, people from ethnic minorities in England, or care home residents being underrepresented [11, 32, 56]. These groups all face specific challenges with regard to LTCM both in general and in the context of HIV, and any further work in this field should ensure greater focus on them to ensure equity and applicability of recommendations.

Despite these limitations, this review provides an inclusive overview of the literature as well as providing grounds for future research and information for commissioners.

## CONCLUSIONS

We present a comprehensive review of the literature on what services, interventions, and support are available in England for People with HIV to manage their overall health and wellbeing. It demonstrates a scarcity of high‐level evidence. Future work could include the development of a toolkit to support commissioners on how to develop and evaluate existing services or commission new ones to address LTCM in people living with HIV. The results could also support guidelines on the management of long‐term conditions in the context of HIV.

## AUTHOR CONTRIBUTIONS

HTJ was responsible for the search strategy, data extraction, analysis, and drafting of the manuscript. TJB aided HTJ with screening of the results for inclusion. All authors contributed to editorial changes of content in the manuscript with all approving the final manuscript. All authors have participated sufficiently in the work and agreed to be accountable for all aspects of the work.

## CONFLICT OF INTEREST STATEMENT

HTJ has received speakers fees from ViiV. TJB has received speaker fees, conference support, and advisory board honoraria from Gilead Sciences LTD., ViiV, Roche, MSD, and Thera. LW has received speaker/advisory fees from ViiV. MSD, and Janssen, and is an investigator on trials sponsored by Gilead, ViiV, and MSD. LL, MR, DA, AW, and IW have no disclosures.

## APPENDIX 1 Search Strategy

((((service* OR intervention* OR support*) AND (UK OR United Kingdom OR England)) AND (HIV* OR AIDS OR human immunodeficiency virus* OR acquired immunodeficiency syndrome)) AND (chronic ill* OR chronic disease* OR long‐term condition* OR long term condition*))

## APPENDIX 2 Data Extraction Table

**Table:**

Title	Authors	Year of Publication	Publication Type	Location	Aim	Sample	Study design	Themes Identified	Key Findings
Patient experience with HIV specialist services and GP services amongst adults living with HIV in England and Wales [14]	Auzenbergs et al.	2018	Conference Abstract	National	To determine patient satisfaction with HIV and GP services for people living with HIV	4415 Adults living with HIV Demographics not stated	Cross sectional Questionnaire (Positive Voices)	HIV services Primary care	Mean HIV clinic rating was 9.3/10 Mean GP rating was 6.9/10 High satisfaction with HIV services GP ratings similar to those given by the general population
Evaluation of services for People with HIV/AIDS in an inner‐city health authority: perspectives of key service providers [15]	Beedham and Wilson‐Barnett	1993	Research Paper	London	To gather information on the social and health care needs of People with HIV from the perspectives of (a) key service providers (b) clients and (c) informal carers	47 Service providers Demographics not stated	Qualitative Semi‐structured interviews	Primary care	Perceived lack of knowledge of HIV amongst GPs, community nurses and community mental health staff Feeling these groups are unwilling to support people living with HIV
Crossing the Divide: Transition Care Services for Young People with HIV—Their Views [16]	Bundock et al.	2011	Research Paper	London	To establish the satisfaction and preferences of young adults transitioning into standard adult HIV services	Total: 50 UK: 21 Adolescents living with HIV Median age 19 Range 17–21 43% Male 33% White	Cross sectional Questionnaire	HIV services	HIV services should be equipped to support adolescents transitioning into adults services and with LTCM
Specialist care of older adults with HIV infection in the UK: a service evaluation [17]	Cresswell and Levett	2017	Research Paper	National	To describe how widespread adapting services to manage age‐related co‐morbidities is and what form such services take	102 HIV clinics 57% Hospital based	Cross sectional Questionnaire	HIV services Primary care Specialist services	Two specialist services existed Most HIV clinics hold a complex patient MDT Many clinics lack allied health care professional input Most clinics refer to external existing clinics or GP for LTCM 23% wanted a specialist ageing clinic; 64% did not; 13% uncertain
Use of the wellness thermometer to improve consultations for patients with human immunodeficiency virus [18]	Croston et al.	2017	Research Paper	Manchester, East Sussex and Milton Keynes	To evaluate the effectiveness of using the wellness thermometer in healthcare consultations with patients with HIV	Total: 238 Adults living with HIV: 231 Service providers: 7 Demographics not stated	Cross sectional Questionnaire	HIV services	Use of a wellness thermometer can help improve consultations by identifying issues with LTCM
GPs' perceived barriers to their involvement in caring for patients with HIV: A questionnaire‐based study [19]	Defty et al.	2010	Research Paper	South of England	To explore GPs' current perceptions of barriers to their involvement in managing patients with HIV	124 GPs Demographics not stated	Cross sectional Questionnaire	Primary care	93% were comfortable with LTCM for people living with HIV 60% felt they not specialists should manage hypertension, diabetes mellitus and hyperlipidaemia 67% felt that people living with HIV wanted their HIV care to be coordinated by HIV services 20% felt people living with HIV want all their care to come from HIV services including LTCM
Primary care consultations and costs amongst HIV‐positive individuals in UK primary care 1995–2005: a cohort study [20]	Evans et al.	2009	Research Paper	National	To describe patterns of consultation and morbidity in primary care and associated costs, amongst people living with HIV with a view to informing the development of integrated care	2189 Adults living with HIV 66% Male	Retrospective cohort Data from UK General Practice Research Database	Primary care	Consultation rates with GPs declined to rates similar to the wider population due to increased access to ART. 5.0 consultations per year by men and 7.3 consultations/per year by women in 1995 4.2 consultations per year by men and 5.2 consultations/per year in women in 2005
Resource implications for oral care of patients with HIV [21]	Gallagher et al.	1998	Research Paper	National	To obtain the views of people living with HIV towards their dental health and dental care provision	69 Adults living with HIV Age 18–64 93% Male	Qualitative Semi‐structured interviews	Primary care	74% of changed their dentist after HIV diagnosis 40% currently registered 45% had withheld their HIV status 25% had been refused dental care Fear of discrimination Preference for hospital dental clinics over community ones
Patients' attitudes to the summary care record and HealthSpace: qualitative study [22]	Greenhalgh et al.	2018	Research Paper	England, Not specified	To document the views of patients and the public towards the summary care record with a particular focus on those with low health literacy, potentially stigmatizing conditions, or difficulties accessing health care.	Total: 170 Adults living with HIV: 11 Age 25–50 55% Male 45% White	Qualitative 103 Semi‐structured interviews and 7 focus groups 1 Focus group with Adults living with HIV	Primary care	The summary care record helps to reduce stigma by not having to inform GPs of HIV status leading to better care coordination and LTCM
HIV in later life: audit findings from a new virtual clinic service for older HIV patients [23]	Gwyn et al.	2018	Conference Abstract	London	To review the results of the HIV in Later Life Virtual Clinic (HILLVC) at Homerton Hospital	118 Adults living with HIV Median age 55 Range 50–85 58.7% Male 7.6% White	Cross sectional Medical records review	Specialist services	Clinic improved the CVD risk assessment from 49.2% to 93.2%. A small number identified as eligible for TDF to TAF switch Few patients had a FRAX or DEXA scan at baseline 30.5% met criteria for DEXA
What is the role of general practice and the potential barriers in providing shared care for people living with HIV: A systematic review [24]	Hawkins and Cassell	2015	Conference Abstract	National	To assess the evidence on the provision and quality of shared care for people living with HIV	Not specified GPs Demographics not stated	Systematic Review	Primary care	81–89% people living with HIV were registered with a GP 78% had shared their status. Most GPs want shared care Barriers: lack of knowledge, accessibility, issues of confidentiality, stigma, and poor communication between services. GP engagement was associated with their experience with HIV, local prevalence and patient level of morbidity
What problems associated with ageing are seen in a specialist service for older people living with HIV? [25]	Jones et al.	2022	Research Paper	London	To identify what problems associated with ageing were seen in a specialist service for older people living with HIV?	35 Adults living with HIV Median age 69 Range 53–93 77% Male 63% White	Cross sectional Medical records review	Specialist services	83% were frail Eighteen issues linked to ageing were identified The most common being affective symptoms (51%), memory loss (37%) and falls (29%)
Met and unmet health, welfare and social needs of people living with HIV [26]	Kall et al.	2018	Conference Abstract	National	To estimate the met and unmet service needs of people accessing HIV specialist services in the UK	4415 Adults living with HIV Demographics not stated	Cross sectional Questionnaire (Positive Voices)	HIV services Peer support	Areas of need particularly treatment advice (61%), information on living with HIV (47%) and adherence support (40%) with >90% needs met. 32% needed peer support/social contact with People with HIV with 56% receiving this 42% needed LTCM with 67%, having needs met The greatest area of unmet need (62%) was for social and welfare services
My HIV Care – Preferences of people living with HIV for medicine‐related support from community pharmacists [27]	Katusiime et al.	2022	Conference Abstract	National	To understand the preferences and perceptions of people living with HIV towards community pharmacist involvement in their care	144 Adults living with HIV Age 22–80 57.5% Male 88.9% White	Cross sectional Questionnaire	Primary care	Community pharmacists (CPs) least helpful for medicine support (2.7%) vs. GPs (13.7%), HIV nurses (35.6%), HIV pharmacists (42.5%), HIV consultants (56.2%) 56.1% unsure or did not want CP involved in their HIV care. Barriers: lack of HIV knowledge, stigma, confidentiality
Participation, roles, and the dynamics of change in a group‐delivered self‐management course for people living with HIV [28]	Kennedy and Rogers	2007	Research Paper	England, Not specified	To examine the accounts people provide about illness management prior to the course, to observe the ways in which people structure their interaction with one another during the course, and to examine the accounts people provide after the course concerning its impact	14 Adults living with HIV Demographics not stated	Qualitative Documentary analysis, pre‐course Interviews, participant observation, and post‐course interviews	Self‐management	The relationships with the host organization and other attendees might be as important in bringing about improvements in self‐management than the content of a course
Learning from the experiences of People with HIV using general practitioner services in London: a qualitative study [29]	Keogh et al.	2016	Research Paper	London	To explore the experiences of People with HIV using general practitioner services in order to identify barriers to use	51 Adults living with HIV Median age 44 Range 18–67 71% Male 57% White	Qualitative 8 Focus Groups (2 MSM; 3 Black African; 2 co‐morbidities; 1 mental health)	Primary care	Presence of co‐morbidities was strongly associated with GP use GPs lack HIV knowledge; new problems automatically attributed to HIV Lack of continuity of care in Primary Care; see same HIV doctor but different GP Lack of communication between GP and HIV team
The role of the general practitioner in the community care of People with HIV infection and AIDS: a comparative study of high‐ and low‐prevalence areas in England [30]	King et al.	1998	Research Paper	London and Nottingham	To examine the role of general practice in areas of England with low and high HIV prevalence and to compare barriers with general practice care in each area.	Total: 647 Cross sectional: Service providers: 411 Demographics not stated Qualitative: 236 Adults living with HIV: 89 Service providers: 147 Interviews: Mean age 34 80% Male 85% White	Mixed methods Cross sectional: Questionnaire Qualitative: 4 Focus Groups (2 people HIV, 2 service providers) 206 interviews (74 people HIV, 132 service providers)	Primary care Palliative care Peer support	Service providers felt general medical care, psychological support, and referrals are important in general practice management of HIV infection. No doctor and only 3% of practice nurses mentioned palliative care as an important role GPs felt they provide one‐to‐one care, local knowledge, 24‐h availability, and a more ‘normal’ environment than the hospital. People living with HIV felt GPs could provide general medical and psychological care Barriers: Lack of knowledge, stigma
Social determinants of health and long‐term conditions in people of Black African and Black Caribbean ethnicity living with HIV in London: A qualitative study [31]	Kolodin et al.	2024	Research Paper	London	To explore the lived experiences of long‐term conditions amongst people living with HIV of Black African and Black Caribbean ethnicities, and how they are impacted by social determinants of health	Total: 24 Adults living with HIV: 20 Service providers: 4 Age 35–65 45% Male 0% White	Qualitative: 4 Focus Groups of Adults living with HIV 4 interviews with service providers	Peer support Primary care Secondary care Self‐management	Diet and exercise noted as important but difficult due to cultural traditions and financial constraints Community organizations' support improved LTCM Stigma is a barrier to primary and secondary care
Access to dental care for HIV patients: does it matter and does discrimination exist? [32]	Levett et al.	2009	Research Paper	London	To assess dental care access amongst people living with HIV and people at risk of HIV acquisition	Total: 241 GU clinic attendees: 190 Adults living with HIV: 51 Median age 43 Range 31–67 58.8% Male	Cross sectional Questionnaire	Primary care	People living with HIV have more difficulty registering with a dentist than seronegative people (58.8% vs. 18.2%) Also have a higher rate of dental health problems (54.9% vs. 32.1%) 6.2% refused dental care 34.6% felt sharing their status had negatively impacted their care
Evaluation of a Combined HIV and Geriatrics Clinic for Older People Living with HIV: The Silver Clinic in Brighton, UK [33]	Levett et al.	2020	Research Paper	Brighton	To evaluate a combined HIV and Geriatrics clinic for older people living with HIV	52 Adults living with HIV Median age 67 Range 53–87 90% Male 96% White	Cross sectional Medical records review	Specialist services	Frailty and low mood common Interventions: medication review, management of geriatric syndromes, referrals to rehabilitation and social services Well received by attendees
The prevalence of comorbidities amongst people living with HIV in Brent: A diverse London Borough [34]	Lorenc et al.	2014	Research Paper	London	What associated comorbidities are present in people in Brent (London, UK) living with HIV, and how common are they?	982 Adults living with HIV Mean age 45 55% Male 23.2% White	Cross sectional Medical records review	Primary care	29% people had one long‐term health problem other than HIV Hepatitis, mental health and cardiovascular disease being most common Primary Care well placed for LTCM as collaborative, holistic, person‐centred and individualized
Shared care: how can we do it? Findings from the BHIVA primary care project [35]	Maclellan et al.	2017	Report	National	To inform commissioning and delivery of high‐quality healthcare for people living with HIV between primary and specialist care across the life course	Cross Sectional Total: 319 GPs: 152 Adults living with HIV: 167 Average age 48 Range 26–72 64% Male 54% White Qualitative Total: 78 Service Providers: 65 People living with HIV: 13	Mixed methods Cross sectional: 2 Questionnaires GPs and Adults living with HIV Qualitative: 65 interviews with Service Providers 1 Focus group with Adults living with HIV	HIV services Primary care	GPs lack experience, time and inclination to support with LTCM Barriers: time, knowledge of the person, HIV, and drug–drug interactions People wanted to access GP in the HIV clinic The average number of people living with HIV on the practice list was 35 (0–150), average prevalence of 3.5/1000 Only 47% registered with a GP High rates of anxiety & depression 68% felt GP was confidential 40% felt GPs gave them enough time Over half unable to see same GP Biggest strength of GP care is their locality GPs: top model was educational updates then shared care
Routine monitoring and assessment of adults living with HIV: results of the British HIV Association (BHIVA) national audit 2015 [36]	Molloy et al.	2017	Research Paper	National	To assess adherence to British HIV Association guidelines for the routine investigation and monitoring of adults living with HIV‐1	8258 Adults living with HIV 66.4% Male 58.8% White	Cross sectional Medical records review	HIV services	HIV services are doing LTCM with focus on cardiovascular and bone health
The provision of dental treatment for HIV positive patients in Manchester [37]	Nazir et al.	2005	Research Paper	Manchester	To investigate the provision of dental treatment for HIV positive patients in Manchester, based upon their personal experiences.	81 Adults living with HIV Mean age 40.5 Range 21–70 76% Male 84% White	Cross sectional Questionnaire	Primary care	75% registered with a dentist Registration and attendance rates increased after diagnosis 64% had shared their status 4% had been refused treatment 68% changed dentist post HIV Barriers: Fear of treatment refusal (50%) and confidentiality (72%). 36% preferred specialist clinic
Whose stable infection is it anyway? Patient and staff perspectives of HIV as a stable condition [38]	Nixon et al.	2015	Conference Abstract	Brighton	To explore the perceived needs of People with HIV and the relationship with processes of care	Total: 34 Adults living with HIV: 13 Service providers: 21 Demographics not stated	Qualitative Semi‐structured interviews	HIV services	People with minimal illness preferred virtual models of care People with higher illness experience more reliant on face‐to‐face interactions Those with uncertainty about their future health or had a negative social identity had a stronger attachment to HIV services
Patient experiences of a community HIV clinic: A qualitative evaluation [39]	Nyabadza and Vilar	2020	Conference Abstract	Manchester	To explore patients' experiences of attending community based HIV satellite clinics	8 Adults living with HIV Demographics not stated	Qualitative Semi‐structured interviews	HIV services	Attending the co‐located clinic improved LTCM Reduction in stigma and treatment burden. Easier to access and more convenient Improved service user‐service provider relationships
Advancing research and practice in HIV and rehabilitation: A framework of research priorities in HIV, disability and rehabilitation [40]	O'Brien et al.	2014	Research Paper	International with UK component but not quantified	To identify current and emerging issues in HIV, disability and rehabilitation	Total: 92 Mix of Adults living with HIV and Service Providers UK: 9 Demographics not stated	Qualitative Multi‐stakeholder multi‐method international consultation	Self‐management	Need more focus on LTCM Need for improved access to rehabilitation for people living with HIV to allow for self‐management of their long‐term conditions
Research priorities for rehabilitation and ageing with HIV: A framework from the Canada‐International HIV and Rehabilitation Research Collaborative (CIHRRC) [41]	O'Brien et al.	2020	Research Paper	International with UK component but not quantified	To identify research priorities in HIV, ageing and rehabilitation	Total: 69 Mix of Adults living with HIV and Service Providers UK: 5 Demographics not stated	Qualitative Multi‐stakeholder multi‐method international consultation	Self‐management	Recognizing that goals, values, preferences and expectations may differ depending on age and length of time living with HIV Self‐management strategies Rehabilitation Interventions that address the multidimensional and episodic nature of disability can reinforce and promote self‐management Goal setting, promoting independence, enhancing recovery, recognizing progress, and valuing reassurance from others are key aspects of self‐management
‘We never expected this to happen’: narratives of ageing with HIV amongst gay men living in London, UK [42]	Owen and Catalan	2011	Research Paper	London	To identify factors that impact on the experience of ageing with HIV in gay men living in London and to identify issues relevant to the provision of health and social care	10 Men living with HIV Median age 60 Range 52–78 100% Male 90% White	Qualitative Semi‐structured interviews	Peer support	Peer support is important in preventing loneliness and social isolation
The last resort would be to go to the GP’. Understanding the perceptions and use of general practitioner services amongst People with HIV/AIDS [43]	Petchey et al.	2000	Research Paper	England, Not specified	To investigate their perceptions and use of general practitioners, with the intention of understanding them in the context of their broader care choices and as components of their overall strategies for coping with HIV infection.	20 Adults living with HIV 90% Male	Qualitative Semi‐structured interviews	HIV services Primary care	All registered with a GP Totality of care by HIV services was preferred Some LTCM better done by GPs Shared care leads to fragmentation Barriers to GP care: lack of HIV knowledge, stigma HIV services were more empathetic and better placed to manage the psychological conditions associated with HIV compared with Primary Care
Patients' preferences for the delivery of HIV services: A qualitative study of the roles of secondary versus Primary Care [44]	Pollard et al.	2015	Conference Abstract	Southeast England	To examine patients' preferences for the future delivery of HIV services	74 Adults living with HIV 61% Male	Qualitative 12 Focus Groups	HIV services Primary care	Barriers to GP care: lack of HIV knowledge, stigma Poor communication between services Preference for maintaining all care within HIV clinics amongst people with longer histories of HIV and/or comorbidities. Unclear who had responsibility for prescribing, referrals and care‐coordination. The value of electronic patient records to facilitate communication was balanced against risks to confidentiality
Integral involvement of People with HIV throughout service design improves usefulness and acceptability [45]	Power et al.	2011	Conference Abstracts	National	To develop a website for people living with HIV to manage their long‐term health	Not specified Demographics not stated	Cross sectional Questionnaire	Self‐management	Comprehensive and interactive online information and services can support LTCM
Planning for end‐of‐life care within lay‐led chronic illness self‐management training: The significance of ‘death awareness’ and biographical context in participant accounts [46]	Sanders et al.	2008	Research Paper	National	To provide a deeper understanding of the lives of people living with a chronic condition and the impact of the EPP (the Expert Patients Programme)	12 Adults living with HIV Demographics not stated	Qualitative Semi‐structured interviews	Palliative care Peer Support Self‐management	Peer support important for LTCM People living with HIV are a heterogeneous group, so interventions need to be aware of this to avoid stigma Palliative care services can support people living with HIV in managing their health e.g. with advanced care planning but this needs to be done sensitively at an appropriate time Self‐management can be undertaken leading to reduced use of healthcare
Healthcare needs beyond HIV in a changing NHS: Participant exploration of Positive Voices data [47]	Smithson et al.	2019	Conference Abstract	National	To explore the long‐term condition management needs of people living with HIV and to establish how these are managed within the NHS, stigma and other barriers to care	28 Adults living with HIV Demographics not stated	Qualitative 4 Focus Groups	HIV services Secondary care Self‐management	High levels of satisfaction with HIV clinics (average rated 9.3/10) 95% felt involved in decisions about their HIV care and enabled to self‐manage their HIV 77% reported other health needs not related to HIV, but only half had these met Difficulty in accessing services and navigating NHS Non‐HIV clinicians lack knowledge or confidence Feel responsible for educating non‐HIV clinicians and researching drug interactions Anticipated or experienced stigma was a key issue (35%) 14% had experienced discrimination in healthcare 11% had been denied or refused a treatment or procedure
Identifying multimorbidity clusters with the highest Primary Care use: 15 years of evidence from a multi‐ethnic metropolitan population [48]	Soley‐Bori et al.	2022	Research Paper	London	To assess the association between multimorbidity clusters and Primary Care consultations over time	Total: 826166 ‘Dependence’ category including HIV: 3299 Mean age 41.7	Retrospective cohort Medical records review	Primary care	People living with HIV were in the group with the highest increases in Primary Care demand as additional long‐term conditions develop
A retrospective review of admissions‐what can we learn? [49]	Sornum et al.	2016	Conference Abstract	London	To delineate the HIV inpatient cohort of our inner‐city London hospital through a retrospective review of all inpatient adult admissions.	99 Adults living with HIV Median age 46 Range 18–84	Retrospective cohort Medical records review	HIV services	26% of admissions were related to LTCM
Frailty and frailty screening: A qualitative study to elicit perspectives of People living with HIV and their healthcare professionals [50]	St Clair Sullivan et al.	2023	Research Paper	Brighton	To explore the perceptions of older people living with HIV and those of HIV professionals towards frailty and routine screening for frailty	Total: 57 Service providers: 12 Adults living with HIV: 45 Median age 59 Range 50–81 64% Male 73% White	Qualitative 45 interviews with Adults living with HIV 2 Focus group with Service Providers	HIV services	Preference for frailty screening and assessment to be done within their HIV service Professionals expressed concerns around identifying frailty in younger patients and the lack of services to manage it Professionals acknowledged that the screening tools were a starting point, overall they had more negative views of them, with some feeling they were not relevant to their cohort or took too long to complete.
A dedicated clinic for HIV‐positive individuals over 50 years of age: a multidisciplinary experience [51]	Waters et al.	2012	Research Paper	London	To describe the development of this service [over 50's clinic at Chelsea and Westminster hospital), our current clinic protocols and a summary of selected service outcomes following the introduction of enhanced physical and psychological screening.	150 Adults living with HIV Median age 58 Range 50–88 93% Male 89% White	Cross sectional Medical records review	Primary care Specialist services	HIV physician led specialist services can be successful in LTCM especially cardiovascular and bone health Service users felt was more holistic than GP care
Social networks, social support, health and HIV‐positive gay men [52]	White and Cant	2003	Research Paper	London	To explore the experiences of social support on the part of a number of HIV‐positive gay men	30 Men living with HIV Mean age 38 Range 25–63 100% Male	Qualitative Semi‐structured interviews	Peer support	Peer support is important to support LTCM
How activated are you? Improving people with HIV's confidence in managing their health [53]	Yong et al.	2018	Conference Abstract	London	To ascertain the feasibility and experience of implementing the Patient Activation Measure (PAM) tool in an inner city HIV service	203 Adults living with HIV Demographics not stated	Cross sectional Questionnaire	Self‐management	Most participants were able to complete the PAM tool Nearly two‐thirds had scores in the most activated range Amongst the least activated were a significant number with mental health problems
